# Effects of the transobturator tape procedure on overactive bladder symptoms and quality of life: a prospective study

**DOI:** 10.1590/S1677-5538.IBJU.2019.0158

**Published:** 2019-12-17

**Authors:** Salih Polat, Tarik Yonguc, Serkan Yarimoglu, Ibrahim Halil Bozkurt, Ertugrul Sefik, Tansu Degirmenci

**Affiliations:** 1 Department of Urology, Amasya University Faculty of Medicine, Amasya, Turkey;; 2 Department of Urology, University of Health Sciences Izmir Bozyaka Education and Research Hospital, Izmir, Turkey

**Keywords:** Suburethral Slings, Urinary Bladder, Overactive, Quality of Life, Urinary Incontinence

## Abstract

**Introduction:**

This study aimed to evaluate the effects of transobturator tape (TOT) on overactive bladder (OAB) symptoms and quality of life.

**Materials and Methods:**

Patients with stress-predominant mixed urinary incontinence (MUI) who had undergone TOT procedures were considered candidates for this research. Preoperative assessment included anamnesis, pelvic examination, cough stress test (CST), and validated symptom severity and quality of life (QoL) questionnaires. The primary outcome, improvement and cure rates of OAB symptoms were determined based on the patient's baseline scores in symptom-related questions in OAB-V8. Secondary outcomes included the success rates of SUI, changes in the QoL score and patient satisfaction rates.

**Results:**

A total of 104 patients were included in the study. Sixty-two patients underwent TOT placement alone, and 42 patients underwent TOT placement along with prolapse surgery. The mean follow-up period of the patients was 30.47 months range: 13-52 months. At the first-year follow-up, 52 patients (50.0%) and 59 patients (56.7%) reported cure in preoperative urgency and urgency incontinence, respectively. The objective and subjective cure rates were 96.2% and 56.7%, respectively. A total of 80.7% of the cases had a 15-point improvement in QoL scores.

**Conclusions:**

MUS is not only a gold standard treatment in SUI but also presents as a promising treatment modality in stress-dominant MUI. Although the improvement rates of OAB symptoms significantly decrease over time, QoL and patient satisfaction rates remain higher than any other treatment in this patient group at the third-year follow-up.

## INTRODUCTION

Mixed urinary incontinence (MUI) is the second most common type of urinary incontinence and is defined as a combination of stress urinary incontinence (SUI) and urgency urinary incontinence (UUI) (1, 2). In many cases, detailed anamnesis shows that SUI is not the only symptom in patients with incontinence. In addition to SUI, these patients can exhibit one or more overactive bladder (OAB) symptoms, such as urgency, UUI, nocturia, and frequency. These patients often report more troublesome symptoms regarding their quality of life (QoL) compared to patients with other types of urinary incontinence (3, 4). Moreover, there is evidence that women with MUI have reduced work productivity, and, in severe cases, urinary incontinence can lead to social isolation (5).

MUI has been a difficult condition for clinicians to treat due to the need for the treatment of both SUI and UUI simultaneously (1). Initially, clinicians believed that surgical treatment was inadequate and that it could even aggravate symptoms (urgency, UUI) in patients with MUI (6). To date, studies have demonstrated variable UUI cure rates, ranging from 40-100% following different midurethral sling (MUS) types in MUI (7, 8). In a recent study that compared the three-year outcomes of two MUS types, the cure rates were reported to be 50% and 56% for urgency and UUI, respectively (9).

Although various studies have evaluated MUS outcomes in women with MUI, these were mostly heterogeneous studies involving different MUS types. Additionally, they lacked mid-term results (10–14). Moreover, these studies did not use validated questionnaires to assess the changes in the patient's overactive bladder symptoms and QoL. To close the gap in the literature, we aimed to evaluate the short- and mid-term effects of outside-in transobturator tape (TOT) procedures on OAB symptoms and QoL of women with MUI.

## MATERIALS AND METHODS

**Figure 1:**
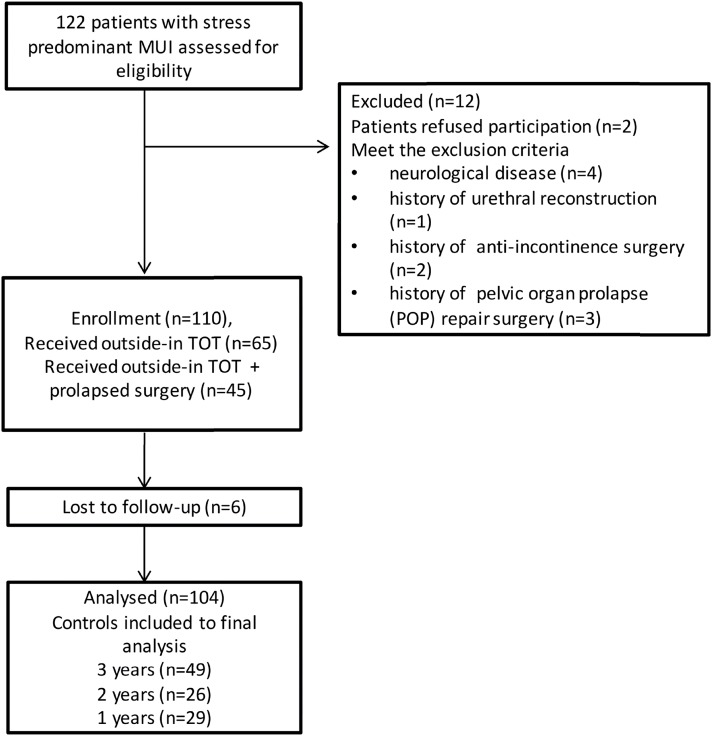
Flowchart of patient selection.

This a prospective study evaluating the short- and mid-term outcomes of outside-in TOT procedures in patients with stress-predominant MUI. The study was conducted between October 2012 and February 2016. The study was approved by the local ethics committee in October 2012. (Decision No: 1, Bozyaka Research and Training Hospital). The participants were informed about the study, and their informed consent was obtained. The patients with stress-predominant MUI who had undergone TOT procedures were considered as candidates for this research. A total of 104 patients were included in this study after the following exclusion criteria were applied: having a neurological disease, history of urethral reconstruction, anti-incontinence and/or pelvic organ prolapse (POP) repair surgery, and being aged below 18 years (Figure-1). The mesh used in the study was composite macropore monofilament polypropylene mesh (Duzey SVT vaginal tape system, Istanbul, Turkey). All operations were undertaken by two experienced surgeons.

### Preoperative and postoperative assessments

The preoperative assessment included a detailed anamnesis, pelvic examination, cough stress test (CST), measurement of post-void residual urine volume, urinalysis and questionnaire forms. The incontinence and OAB symptom severity of each patient was measured using the International Consultation on Incontinence Questionnaire-Short Form (ICIQ-SF), Urinary Distress Inventory-6 (UDI-6), and Overactive Bladder Awareness Tool Version 8 (OAB-V8). QoL was evaluated for all patients based on the stress- related leak, emptying ability, anatomy, protection, and inhibition (SEAPI) scoring system and Incontinence Impact Questionnaire-7 (IIQ-7). The validated Turkish versions of these questionnaires were used. The presence of OAB symptoms, such as frequency, nocturia, urgency and UUI, and demographic data, such as parity, body mass index, hysterectomy history were noted. CSTs were performed while the patients were in a lithotomy position and had bladder volumes of 300mL. The results of the CSTs were reported as positive if urine loss occurred when coughing. If no urine loss was observed, CST was repeated with the patient in a standing position.

The baseline evaluation was comprised of patient's medical history, general examination and pelvic examination, and the questionnaires were administered both at the baseline evaluation and postoperative month 3 and years 1, 2 and 3. In order to observe the effect of the operation, the patients were asked to stop their anticholinergic medicine before the baseline evaluation and each follow-up session. The primary outcome, cure and improvement rates of OAB symptoms were determined based on the patient's baseline scores in symptom-related questions in OAB-V8, and the cure was defined as a postoperative response of 0. Clinical improvement was defined as a postoperative reduction in the scores, given a baseline score of greater than 0. Persistence was defined as a postoperative score equal to that recorded at the baseline and worsening as a postoperative score greater than the baseline score. Secondary outcomes and improvement rates of SUI and QoL were measured as follows: cure of incontinence was defined as being completely dry after surgery, which was assessed subjectively and objectively. The criteria for objective cure of SUI included a negative CST, no need for pads, and requirement of no additional operation for SUI. The subjective cure was defined as an ICIQ-SF score of 0 and no need for pads. It was also analysed whether subjective failure was due to stress, urge, or mixed incontinence (item 6, ICIQ-SF). A clinical improvement in QoL was defined as a reduction of at least 15 and 7 points compared to the patient's baseline SEAPI and IIQ-7 scores, respectively (15, 16). The postoperative satisfaction of the patients was assessed using a Visual Analogue Scale (VAS), in which a value of 0 indicated ‘not at all satisfied’, and a value of 100 indicated ‘very satisfied’. The patients with scores of 80 or above were considered to be satisfied with the operation.

#### Statistical analysis

The Shapiro-Wilk test was used to determine whether the distributions of continuous variables were normal. The continuous data were presented as mean±standard deviation (SD). The mean differences between two related groups of normally distributed data were compared with a paired-samples t-test, while the Wilcoxon T-test was used to compare the non-normally distributed data. Repeated measures for ANOVA was used to compare more than two related groups. The rates of satisfaction and clinically improvement in QoL at the third-year follow-up were compared to those of first-year and second-year follow-ups using McNemar's test using the Bonferroni-corrected significance level of 0.017.

## RESULTS

In this study, 62 patients underwent TOT placement alone, and 42 patients underwent TOT placement along with prolapsed surgery (POPQ stage 2 and above). Anterior colporrhaphy (AC) was performed in 17 patients, AC with mesh in 11 patients, posterior colporrhaphy (PC) in eight patients, AC+PC in four patients, and sacrospinous fixation in two patients. The demographic data of the patients are shown in Table-1. The mean age of the patients was 53.04 years, ranging from 27 to 78 years. The mean follow-up period was 30.47 months (range: 13-52 months). The objective cure rates of SUI were calculated as 96.2% at 3 months, and decreased from 95.2% to 79.6% over the three year follow-up period. The subjective cure rates were 56.7% at 3 months, and decreased from 52.8% to 34.6% over the three years (Table-2). Subjective failure was due to the existence of urgency incontinence in 41 patients (39.4%) and mixed incontinence in four patients (3.8%). The use of anticholinergics was required in 86 patients preoperatively and 43 patients at postoperative month 3.

**Table 1:** Demographic data of the patients.

No. of patients		104	
Median age years(min-max)		53	(27-78)
**Age ranges n(%)**			
	0-50	45	43.3%
	51-60	38	36.5%
	>60	21	20.2%
**Parity n(%)**			
	0	2	1.9%
	1	6	5.8%
	2	42	40.4%
	3	27	26.0%
	>4	27	26.0%
**BMI (kg/m^2^)**			
	<30	47	45.2%
	30-34.9	31	29.8%
	35≤	26	25.0%
Previous hysterectomy n(%)		16	15.4%
Post-menopause n(%)		65	62.5%

**SD** = Standard deviation; **BMI** = Body mass index

**Table 2:** The outcomes of the TOT procedure and the change of overactive bladder symptoms and questionnaire at 3 months, 1,2 and 3 years.

		Pre-op	3 month		P value	1 year		2 years		3 years		P value
ICIQ-SF score		17.1±2.7	4.9 ± 6.2		<0.001*	5.3 ±6.7		7.4 ± 7.6		8.7 ±8.2		<0.001¥
OAB-V8 score		22.7 ± 7.1	10.5 ± 9.1		<0.001†	11.4 ± 9.5		12.3 ±9.9		14.6 ± 9.9		<0.001¥
SEAPI score		28.2 ±8.8	5.8 ±8.7		<0.001*	6.5 ±9.6		8.7 ± 11.2		10.0 ± 11.8		<0.001¥
IIQ7 score		15.0 ±5.2	3.1 ± 4.5		<0.001*	3.4 ±4.9		4.6 ± 5.9		6.5 ±7.1		<0.001¥
Clinically improvement in QoL(SEAPI) (n/total, %)			84/104, 80.7			83/104, 79.8		51/75, 68.0		30/49, 61.2		1. vs 2. yr 0.031# 2. vs 3. yr 0.250#
Postoperative satisfaction rates (n/Total, %)			89/104, 85.5			87/104, 83.6		56/75, 74.6		35/49, 71.4		1. vs 2. yr 0.039# 2. vs 3. yr 0.500#
			**3 months**				**1 year**	**2 years**		**3 years**		
			n	%		n	%	n	%	n	%	
**Outcomes**												
Objective cure			100	96.2		99	95.2	68	90.7	39	79.6	
Subjective cure			59	56.7		55	52.8	38	50.6	17	34.6	
**Symptoms**												
**Urgency**												
	Cured		56	53,8		52	50,0	34	45,3	16	32,7	
	Better		27	26,0		30	28,8	24	32,0	15	30,6	
	Same		16	15,4		16	15,4	12	16,0	11	22,4	
	Worsened		5	4,8		6	5,8	5	6,7	7	14,3	
	Total		104			104		75		49		
**Urgency incontinence**												
	Cured		63	60,6		59	56,7	40	53,3	18	36,7	
	Better		20	19,2		23	22,1	17	22,7	13	26,5	
	Same		17	16,3		18	17,3	14	18,7	11	22,4	
	Worsened		4	3,8		4	3,8	4	5,3	7	14,3	
	Total		104			104		75		49		
**Frequency**												
	Cured		37	43,0		35	40,7	22	34,9	11	27,5	
	Better		30	34,9		30	34,9	26	41,3	17	42,5	
	Same		16	18,6		18	20,9	10	15,9	6	15,0	
	Worsened		3	3,5		3	3,5	5	7,9	6	15,0	
Total			86			86		63		40		
**Nocturia**												
	Cured		21	26,6		20	25,3	16	28,1	9	23,1	
	Better		38	48,1		37	46,8	22	38,6	13	33,3	
	Same		19	24,1		21	26,6	16	28,1	12	30,8	
	Worsened		1	1,3		1	1,3	3	5,3	5	12,8	
**Total**			**79**			**79**		**57**		**39**		

* Wilcoxon test

¥ ANOVA for Repeated Measures

† Paired-Samples T-test

# McNemar test (significant level of 0.017)

ICIQ-SF: International Consultation on Incontinence Questionnaire - Short Form, UDI-6: Urogenital Distress Inventory, OAB-V8: 8-item overactive bladder questionnaire, SEAPI: Incontinence Quality of Life Score questionnaire, IIQ7: The incontinence impact questionnaire-7

### Changes in patients’ OAB symptoms

**Figure 2:**
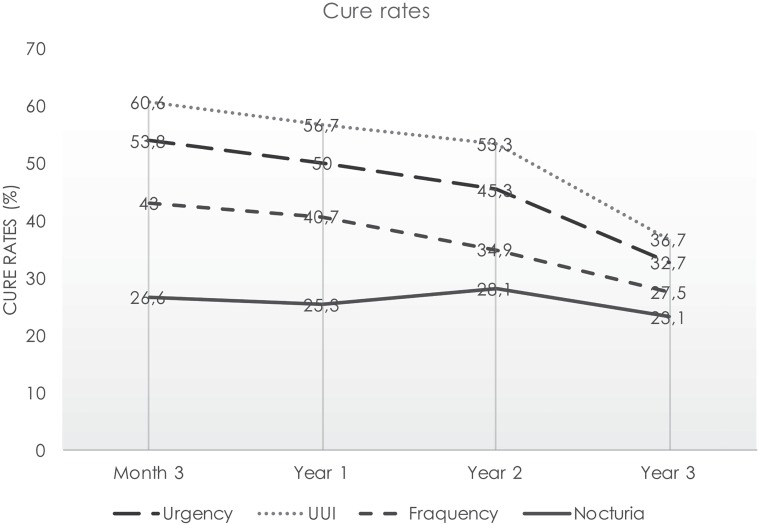
Changes in overactive bladder symptoms over time.

The results of the changes in OAB symptoms are shown in Table-2 and Figure-2. UUI and urgency were present preoperatively in all patients. Frequency and nocturia were present preoperatively in 86 and 79 patients, respectively. The placement of a mid-urethral sling resulted in an overall significant improvement in all OAB symptoms, with the greatest improvement being observed in relation to UUI. At the third postoperative month, 60.6% of the patients were cured of UUI and the cure rate was calculated as 56.7%, 53.3% and 36.7% at postoperative years 1, 2 and 3, respectively. Worsening of UUI was detected in 3.8% of the patients at the third postoperative month, and this rate increased to 14.3% at the end of the third year. For urgency, the cure rate was 53.8% at the postoperative third month, followed by 50.0%, 45.3% and 32.7% at postoperative years 1, 2 and 3, respectively. Worsening of urgency was detected in 4.8% of the patients at the third postoperative month and 14.3% of the patients at the end of the third year. Nocturia disappeared completely only in 26.6% of the patients, with a further 48.1% recording an improvement. At the postoperative third year, the cure and improvement rates decreased to 23.1% and 33.3%, respectively. For frequency, the cure rate was 43.0% at the postoperative third month and was reduced to 27.5% at the end of the third year.

The mean scores of the OAB-V8 questionnaire before and after TOT placement are shown in Table-2. Compared to the baseline, statistically significant improvements were observed in total postoperative OAB scores, ranging from 22.7 to 10.5 (p <0.001). The OAB scores gradually increased over time, and finally at the third year, the mean score was calculated as 14.6, compared to 11.4 at year 1. The increase in the symptom scores was statistically significant (p <0.001).

### Changes in quality of life

The changes in the SEAPI and IIQ-7 scores are shown in Table-2. The mean preoperative and postoperative SEAPI scores were 28.2 and 5.8, respectively (p <0.001). The mean score gradually increased over years, being calculated as 6.5 at year 1 and 10.0 at year 3 (p <0.001). A similar increase was observed in the IIQ-7 score (p <0.001). At the postoperative third month, 80.7% of the patients (84/104) were observed to have significant clinical improvement in QoL. Although there was a decrease in clinical improvement in the following years, no statistically significant change was observed after the Bonferroni correction (significance level 0.017) (p=0.031 for year 1 vs. year 2; p=0.250 for year 2 vs. year 3).

### Changes in satisfaction rates

The patient satisfaction rates were 83.6%, 74.6%, and 71.4% for postoperative years 1, 2 and 3, respectively, and the overall satisfaction rate was 85.5% at the postoperative third month. Although the patient satisfaction rate reduced over years, this was not statistically significant (p=0.017) when the Bonferroni correction was applied (p=0.039 for year 1 vs. year 2; p=0.500 for year 2 vs. year 3).

## DISCUSSION

Urinary incontinence is associated with significant morbidity and can have a considerable impact on QoL (17, 18). Approximately 50% and 30-40% of women with UI have SUI and MUI, respectively (19). Compared to women with other types of urinary incontinence, those with MUI often report that their symptoms are more troublesome (4, 9). To date, two mid-urethral sling techniques have been developed: tension-free vaginal tape (TVT) introduced in 1990 by Petros and Ulmsten (20) and TOT introduced in 2001 by Delorme (21). Since then, SUI has been treated with much success. Many studies have demonstrated that mid-urethral slings also improve the storage symptoms in MUI (7, 22). In the literature, the most significant improvement after TOT has been reported in urgency and UUI. In a review evaluating MUI symptoms after MUS (TOT, TVT, TVT-O), Jain et al. (7) found a 30 to 85% improvement in urgency and UUI during a follow-up of three to 60 months. In a recent study, Natale et al. (23) calculated the rates of postoperative urgency and UUI as 70.9% and 74.4%, respectively. Abdul-Fattah et al. (9), comparing the outside-in TOT and inside-out TOT (TVT-O) operations, reported a cure rate of 52% in urgency and 57.4% in UUI after 12 months of follow-up, suggesting no significant difference between the two methods. Similarly, in our study, the cure rates were determined as 53.8% for UUI and 60.6% for urgency. In two studies evaluating the medium and long term outcomes over three- and nine-year follow-ups, Abdul-Fattah et al. reported a reduction in the cures rates of urgency and UUI, being determined as 51.5% and 57.0%, respectively at year 3 and 35% and 41%, respectively at year 9 (9, 24). Similar results were obtained from the review of Jain et al. (7). Similarly, in the current study, the cure rates were 53.8% for urgency and 60.6% for UUI at the third-month and were found to be reduced to 32.7% and 36.7%, respectively at the end of the third year. In almost all studies involving a medium to long-term follow-up, the cure rates have been observed to decrease over time. Jahanlu and Hunskaar (25) followed up continent women aged 41 to 45 years for 10 years and detected newly developed urinary incontinence in 40%, of whom 18.3% was defined as urgency and 20.3% as mixed-type urinary incontinence. Therefore, it can be stated that the decrease in cure rates is not only affected by the operation but also age. In addition, some studies showed a rapid decrease in cure rates, while others revealed a more stable decrease. This can be explained by multifactorial aetiology-caused MUI or different inclusion criteria (exclude patients with DO) and follow-up protocols of the studies, including the cessation of anticholinergic use before the control visit.

In addition to the symptoms of MUI, recovery and cure rates have also been reported for nocturia and frequency as components of overactive bladder syndrome after TOT. Hensel et al. (26) calculated the cure rates of nocturia and frequency as 22% and 41.5%, respectively in a three-month follow-up. In another study evaluating nocturnal symptoms following outside-in TOT, Lee et al. (27) found a cure rate of 55.7%. In our study, the cure rates were 26.6% for nocturia and 43.0% for frequency. The cure rate of nocturia was lower than those of urgency and UUI, which can be explained by the influence of other pathology-related nocturnal polyuria. In studies investigating the aetiology of nocturia, the authors reported that the lower urinary tract problems was not the major cause of this condition (28, 29).

Unlike the symptomatic healing of OAB symptoms after MUS placement, some patients experience worsening symptoms. Athanasiou et al. (22) assessed the effects of TVT and TVT-O procedures on OAB symptoms, showing that preoperative urgency, UUI, nocturia, and frequency worsened in 18.8%, 10.6%, 24.7% and 16.5% of cases, respectively within the first year. Another study conducted by Hensel et al. (26) also reported that UUI, nocturia, and frequency worsened in 9.7%, 11.3% and 1.9% of their patients at three months after the TVT-O procedure. Our study yielded different results, indicating worsening of urgency in 5.8% of the cases, UUI in 3.8%, nocturia in 1.3%, and frequency in 3.5% at year 1. The worsening rates in our study were lower than those reported by Athanasiou et al. (22) and Hensel et al. (26), which may be related to surgeon experience and our sample consisting of patients with a diagnosis of advanced symptomatic MUI.

Reporting of QoL in clinical studies which assess TOT is limited. In addition, different questionnaires are often used, which makes it difficult to compare the results. Only limited studies have assessed QoL after TOT placement. Habibi et al. (30) found that the beneficial effects of TVT-O placement on QoL persisted at 12 months and that the QoL measurements were correlated with urgency outcomes. In another study which evaluated the long-term effects of TOT and TVT-O on QoL, 85-87% of patients reported significant improvements in QoL (9, 24). In our study, although statistically significant deterioration was observed in QoL scores, the improvement in QoL was maintained in the short- and mid-term after TOT.

Our postoperative patient satisfaction rates were consistent with the literature. Habibi et al. (30) assessed the effects of TVT-O procedures on MUI and reported that 87% of their patients were satisfied at 12 months. Costantini et al. (31), comparing TO-TVT with RP-TVT in an RCT, determined that 31 women (66%) in the TOT group had baseline MUI and showed a patient-reported success rate of 77.4% at year 3. Habibi et al. (30) also reported that patients who were cured of MUI after having incontinence surgery had higher satisfaction rates compared to those with persistent UUI. In our study, the overall satisfaction rate was significantly higher compared to those with persistent UUI at the first- and third-year follow-up (90% versus 56%, p <0.001 at year 1; 82% versus 32%, p <0.001 at year three). These findings highlight the importance of patient counselling for all possible outcomes after sling placement, including a discussion of possible outcomes related to urgency and UUI.

The present study provided a longitudinal, prospective follow-up of different postoperative points to assess the durability of OAB symptoms and QoL outcomes over time using a validated questionnaire assessment. Much of the previous investigation is retrospective and failed to use validated outcome measures or assesses outcomes. A primary limitation of the current study is the inclusion of patients who had undergone concomitant prolapse surgery, which may have affected postoperative outcomes. Another limitation is that the mid-term follow-up included only 49 patients. Despite these limitations, our data contribute to the accumulating literature, which evaluates U/UUI, nocturia, frequency and QoL outcomes after TOT placement.

## CONCLUSIONS

To date, an ideal treatment modality for women with MUI has not yet been described. As shown in the current study, MUS is not only a gold standard treatment in SUI but also presents as a promising treatment modality in stress-dominant MUI. Although the improvement rates of OAB symptoms significantly decrease over time, QoL and patient satisfaction rates remain higher than any other treatment (conservative and pharmacologic) in this population at three-year follow-up. Before TOT procedures, the patients should be informed that OAB symptoms including UUI may persist or even worsen.

## LIST OF ABBREVIATIONS

MUI: Mixed urinary incontinence
SUI: Stress urinary incontinence
OAB: Overactive bladder
UUI: Urgency urinary incontinence
QoL: Quality of life
MUS: Mid-urethral sling
TOT: Transobturator tape
CST: cough stress test
ICIQ-SF: Incontinence Questionnaire-Short Form
UDI-6: Urinary Distress Inventory-6
OAB-V8: Overactive Bladder Awareness Tool Version 8
SEAPI: Each patient's QoL was evaluated using their the stress-related leakage, emptying ability, anatomy, protection, inhibition
IIQ-7: Scoring System and Incontinence Impact Questionnaire-7
VAS: Visual Analog Scale
